# *Trigonella foenum-graecum L.* Sprouted Seed Extract: Its Chemical HPLC Analysis, Abortive Effect, and Neurodevelopmental Toxicity on Mice

**DOI:** 10.1155/2020/1615794

**Published:** 2020-04-07

**Authors:** Sara Oufquir, Mehdi Ait Laaradia, Zineb El Gabbas, Kenza Bezza, Jawad Laadraoui, Rachida Aboufatima, Zahra Sokar, Abderrahman Chait

**Affiliations:** ^1^Laboratory of Pharmacology, Neurobiology and Behavior, Semlalia Faculty of Sciences, Cadi Ayyad University, Marrakech, Morocco; ^2^Laboratory of GénieBiologique, Faculty of Sciences and Technics, Sultan Moulay Slimane University, Béni Mellal, Morocco

## Abstract

The *Trigonella foenum-graecum L*. seeds, in a dormant or sprouted state, have been largely investigated for their therapeutic activities such as being antidiabetic, antioxidant, cholesterol-lowering, and as a digestive enhancer too. Nevertheless, there are no studies evaluating the potential developmental toxicity of germinated grains despite the availability of numerous research studies demonstrating the teratogenicity effect of unsprouted seeds. Therefore, this research work was conducted to assess the impact of fenugreek sprouts on maternal and neurobehavioral developmental toxicities on mice. The lyophilized aqueous extract of germinated seeds was administered via oral gavage on a daily basis to five groups of mated female mice throughout pregnancy at doses of 200, 500, 800, and 1000 mg/kg/day and the control group was administered distilled water. Maternal reproductive toxicity was evaluated, and the surviving pups were assessed for their physical development, malformation, and neurobehavioral toxicity by using a battery of tests from birth to the 25^th^ postnatal day. Additionally, the aqueous extract of germinated and ungerminated seeds was analyzed by high-performance liquid chromatography (HPLC) for a comparison of their major compounds. For pregnant treated female mice, no death and no intoxication symptoms have been registered during the test. However, the sprouts' extract has provoked a significant decrease in fertility, spontaneous abortion, pup's mortality, and neurobehavioral disorder in offspring. HPLC analysis reveals an increase in total phenolic compound concentration by the process of sprouting.

## 1. Introduction

Medicinal plants have been used since the earliest documented history around the world, as an available and inexpensive therapeutic recourse. Statistics show that not less than 80% of the global population continues to benefit from traditional medicine as a primary source for healthcare, for example, Africa, where plants are the most easily accessible and affordable therapy to the local community [1].

Nevertheless, the use of herbal medicine, without any knowledge of their toxicological profile, target organ, and safe dose, is one of the biggest problems of recent healthcare systems. Hence, concern is rising when plants are used by pregnant women, the risk is doubled affecting both the mother and the developing fetus, especially when we know that the prevalence of medicinal plant taken during pregnancy is between 5% and 70% [2–4]. Herbal drugs are used to treat different pregnancy-related problems such as nausea, morning sickness, vomiting, cough, and nutritional deficiencies [5]. However, the effect of such use is still unclear and unsecure and could cause many health disturbances not only in fetal life but at the adult age of progeny.

*Trigonella foenum-graecum L*. or fenugreek belonging to the family of Fabaceae is one of the oldest traditional medicinal plants and it is cultivated in the Indian subcontinent, Mediterranean Europe, and North Africa. This herbal drug has many forms of uses such as dried leaves (herb), spice (seeds), or vegetable (fresh leaves) [6]. Seeds are famous for their spicy aromatic properties and are the most common seasoning product [7]. The study conducted by Ghedira [8] showed that *Trigonella foenum-graecum L.* grains, which have a great nutritional value, were traditionally used for improving the milk production of breastfeeding women. In Morocco, use of seeds is highly recommended for Saharawi women as an appetite stimulant and to improve their physical attractiveness [9]. Additionally, the grains are used as a remedy against stomach disorders, constipation, diabetes, fever, and anemia [10].

Recent scientific research studies have shown that fenugreek has numerous therapeutic benefits such as hepatoprotective, anti-inflammatory, antiulcer, antilithigenic, anticarcinogenic, antibacterial, and neuroprotective effects. Such activities have been proven in both experimental animals and clinical trials on humans [11–14]. Sprouting seeds do not alter their phytotherapeutic activities such as antidiabetic, anticholesterolemic, and antioxidant [15–19].

However, the therapeutic benefits of fenugreek seeds do not make them totally safe. Many studies have reported the toxicological impact of this plant on reproduction. In fact, the study by Al-Yahya [20] demonstrated that fenugreek administration to males mated with untreated females induces teratogenic, foetotoxic, and reproductive changes and the abnormal shapes of the sperms. Other studies show that these seeds have an estrogenic activity that disturbs the endometrial lining system and interferes with fetal development [21, 22]. In addition, fenugreek treatment of pregnant rats caused severe adverse alterations on fetus such as a disorder in developing hind limb long bone [23]. Research work carried out in our laboratory has shown that the seeds' aqueous extract of *Trigonella foenum-graecum* administered orally throughout pregnancy provokes a decrease of fertility on female mice, facial malformation, growth retardation, alteration of motor skills, and neurobehavioral performance in progeny [24–26].

Even though the teratogenicity of ungerminated seeds has been proven by numerous studies, the developmental toxicity of sprouted grains has not been explored yet. There are no data in the literature treating the impact of fenugreek sprouts on reproduction. Therefore, our motivation is to investigate whether the process of sprouting, which provokes a big change in the phytochemical composition of seeds, inhibits or enhances this toxic effect on reproduction.

## 2. Materials and Methods

### 2.1. Plant Material

Organic seeds of *Trigonella foenum-graecum L*. (in Arabic = *El Halba*) were purchased from NATURALTEIN German Company.

Soaked seeds were placed in glass germinators until sprouts were approximately 2 cm. Sprouted and ungerminated seeds were ground into fine powder after drying under the shade at ambient temperature. The powder was extracted with distilled water (1 g/10 ml) under agitation for 12 h. Aqueous extract was centrifuged (1200 rpm), filtered, and lyophilized in Christ apparatus. The extract was then stored in a freezer at −20°C until utilization.

### 2.2. Animals

The present study was conducted in adult *Swiss albino* mice, and female mice (26–28 g) and male mating mice (27–29 g) were used and randomly selected. Female mice were separated from males before starting the test. The mice were acclimatized before and during the experiment, with free access to food and water, and maintained at standardized conditions with a controlled temperature of 23 ± 2°C, a relative humidity of 45–55%, and a 12 h dark/12 h light cycle. Mice were supplied by the Animal Facility of the Semlalia Faculty of Sciences, University of Cadi Ayyad, Marrakesh, Morocco. During all the experiments, tests were carried out in conformity with the basis of approved institutional procedures and the provisions for the care and use of animals as outlined in the Law of 1986 on Scientific Procedures in Live Animals (European Council Directive: 86/609 EEC). Every effort has been made to minimize animal suffering.

### 2.3. Developmental Toxicity

Forty virgin female mice were mated with males previously determined to be fertile (two females and one male in each cage), and the start of pregnancy was confirmed with a vaginal plug, defined as PN0. Then, the female mice were separated in individual cages for oral fenugreek treatment during the entire period of gestation. The pregnant mice were split into five groups of eight animals each. Since the day 0 of gestation, the control group was administered distilled water and the treated groups received 200, 500, 800, and 1000 mg/kg/day of LeGFS.

### 2.4. Maternal Toxicity

Fertility and spontaneous abortion of pregnant mice were observed along the gestation period. The presence of vaginal bloodstains on day 15 of gestation was examined to detect any signs of abortion and/or fetal expulsion. Pregnancy, abortion, and deliverance indexes have been determined as follows:

- Pregnancy index: the percentage of pregnant mice, which have shown a vaginal plug
- Abortion index: the percentage of the number of spontaneously aborted offspring among animals
- Deliverance index: the percentage of female mice delivering among pregnant animals

### 2.5. Postnatal Abnormalities and Viability of Newborn

At postnatal day 0 (PN0), live offspring were examined for external malformations and weighed. Physical developments were noted as follows: hair appearance, incisor eruption, and bilateral eye opening. These parameters are useful indicators of fetal development in mice. Viability and lactation indexes were evaluated on the fourth and the twenty-first days, respectively, as follows:

- Viability index: the percentage of living offspring on the fourth day of lactation among the progeny born alive
- Lactation index: the number of living offspring on day 21 relative to the number of offspring born alive, expressed as a percentage

### 2.6. Neurobehavioral Evaluation of Offspring Mice

#### 2.6.1. Surface Righting Reflex Test

This reflex is the first reaction tested in the rodent after birth. The experiment is to see how neonatal mice in a situation of imbalance could regain their normal quadruped position. The PN3, PN4, and PN5 mice are placed on their back on a soft wooden horizontal plane. The time required to right themselves to a position where all four feet touched the surface was recorded, and the duration of the test is limited to 120 seconds [27].

#### 2.6.2. Cliff Avoidance Test

The offspring were placed on the edge of a table with the forepaws and nose on the border. The time required to finish reversing and turning away from the cliff was recorded. The maximum duration of the test was 120 seconds and conducted on days PN6, PN8, and PN10 [27].

#### 2.6.3. Negative Geotaxis Test

In days PN6, PN8, and PN10, offspring were placed on a surface inclined 45° with their heads facing downward, and the time required to rotate to 90° and subsequently to 180° was recorded [28]. The animals were tested for a duration of up to 120 seconds.

#### 2.6.4. Jumping Down with Choice Test

At PN15 and PN17, the offspring were put on a raised platform 25 cm high above two cages: one with their siblings and the other was empty. The time to jump down into either of the two cages and the choice were recorded [27]. The maximum duration of this test was 120 seconds.

#### 2.6.5. Rotarod Test

Using a rotarod apparatus, motor performance was evaluated, with a rotating rod of 6 cm in diameter (20 RPM). The offspring were tested for their ability to keep their balance on a horizontal rod and to prevent slippage, in one trial per day, for PN23 through PN25. The duration of each animal's stay on the rotating rod was registered; the maximum duration of the test was 5 minutes.

### 2.7. Phytochemical Study

#### 2.7.1. Phytochemical Screening

Preliminary phytochemical screening makes it possible to identify all the chemical families present in LeGFS and involves qualitative determinations of the following substances: flavonoids, tannins, saponins, anthocyanins, leucoanthocyanins, quinones, terpenes, and steroids.

#### 2.7.2. HPLC Analysis

Liquid chromatographic analysis was carried out for both dormant and germinated seeds' aqueous extracts, using HPLC equipped with an automated injector (Shimadzu (Japan) SCL-10A series pumping system, SIL-10AD), UV-visible detector from 200 to 700 nm (SPD 10A), and Shimadzu data software. A separation was performed using Reversed-Phase (RP-18) Columns Agilent Technologies (250 mm × 4.6 mm, 5.0 *μ*m) protected by an Agilent Technologies RP-18 (10 mm × 4.6 mm) precolumn at 25°C with isocratic elution of acetonitrile (5%) and water (95%) in a constant flow rate of 0.1 ml/min, injection volume of 10 *μ*L, and phosphate buffer solution at the pH = 2.6. Phenolic compounds were identified by comparing their retention times with those of standards.

### 2.8. Statistical Analysis

Data are reported as mean ± SEM and were subjected to a one-way test (ANOVA). Differences between group means were tested with the Turkey test. Significance was confirmed with *p* values lower than 0.05. Data analysis was executed using the computer software SigmaPlot 12.5 for Windows.

## 3. Results

### 3.1. Maternal Toxicity

**Figure 1:**
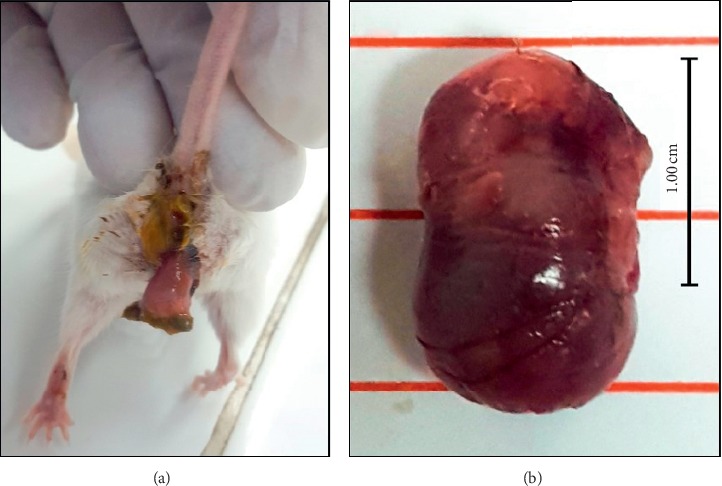
A photo of one of the mice during the gestation period at PN17 aborted following treatment with an aqueous extract of sprouted fenugreek seed (a) and the fetus aborted in postnatal day 17 (b).

In a global evaluation of maternal toxicity, all females survived until study termination, without any abnormal or clinical symptoms, during both gestation and lactation periods. Conversely, as the dose of LeGFS increases, the pregnancy index decreases, especially at D800 and D1000 with 25% loss of fertility. The abortive effect starts from D500 at the percentage of 42.85% and increases to 66.66% at D800 and D1000. In parallel, it is remarkable that abortion in mice took place between gestation days of 16 and 18 (Figure 1).

The deliverance index shows a decrease at high doses of D800 and D1000 where only 33% of female mice ended their gestation period (Table 1).

### 3.2. Postnatal Abnormalities and Viability of Newborn

The morphological exam of newborn has not shown any external malformation, whereas deliverance, lactation, and viability indexes were significantly decreased from D500 to D1000 compared to the control group (*p* < 0.001). D200 does not affect significantly the value of those indexes (Table 2).

Concerning the offspring's body weight at birth, there is no significant difference between control and treated groups. Afterward, low doses reduce significantly the weight of offspring at D200, D500, and D800 during PN3, PN6, and PN9, respectively (*p* < 0.001). Oppositely, we have noticed that at D1000, the LeGFS seems to provoke an increase of weight gain which becomes significant at PN18 and PN21 (*p* < 0.05) (Table 3).

Results summarized in Table 4 show that LeGFS extract delays the development of some morphological facial characters in newly born pups. Actually, the time of incisor eruption, appearance of hair (*p* < 0.001), and opening of the auditory canal (*p* < 0.01 for D200 and D500; *p* < 0.001 for D800 and D1000) are significantly increased by the prenatal exposition, whereas the eye opening is significantly retarded only at high doses of D800 and D1000 (*p* < 0.001) (Table 4).

### 3.3. Neurobehavioral Evaluation of Offspring Mice

#### 3.3.1. Surface Righting Test

**Figure 2:**
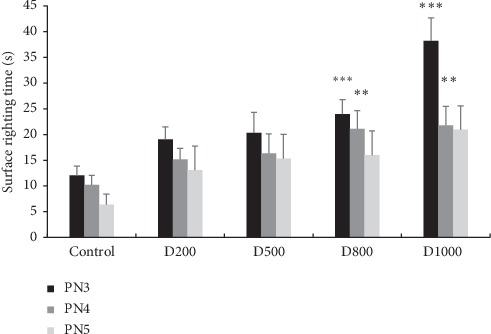
Effects of prenatal treatment with *Trigonella foenum-graecum L.* extract during the entire period of gestation on latencies in surface righting test of 3, 4, and 5 postnatal days. Results are presented as mean ± SEM. ^*∗∗∗*^*p* < 0.001; ^*∗∗*^*p* < 0.01 vs. control (one-way ANOVA). PN: postnatal day; D: dose.

Results presented in Figure 2 show that the turnaround time is increased at all prenatally exposed doses D200, D500, D800, and D1000 and in a significant way at D800 and D1000 on the third day-old (*p* < 0.001) and fourth day-old mice (*p* < 0.01) (Figure 2).

#### 3.3.2. Negative Geotaxis

**Figure 3:**
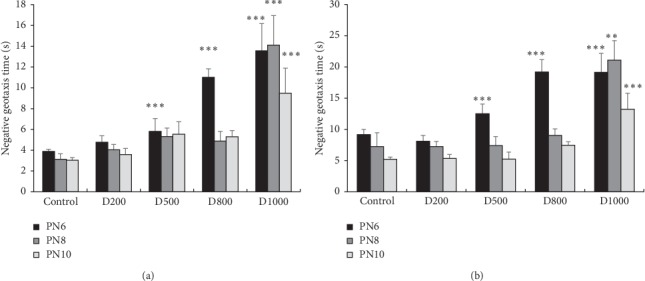
Effects of prenatal treatment with *Trigonella foenum-graecum L.* during the gestational period on latencies in negative geotaxis test: (a) latency to turn to 90° and (b) latency to turn to 180°. Results are presented as mean ± SEM. ^*∗∗∗*^*p* < 0.001; ^*∗∗*^*p* < 0.01 vs. control (one-way ANOVA). PN: postnatal day; D: dose.

Statistical ANOVA tests show that pups pretreated with LeGFS at D1000 take more time to turn and orient their body at 90° and 180° for the 6, 8, and 10 postnatal day-old mice (*p* < 0.001). Significant increase in latency time was equally observed at D500 and D800 (*p* < 0.001), but only at the sixth day-old mice. On the other hand, prenatal administration of D200 of LeGFS seems not to affect offspring performance on the geotaxis test (Figures 3(a) and 3(b)).

#### 3.3.3. Cliff Avoidance Test

**Figure 4:**
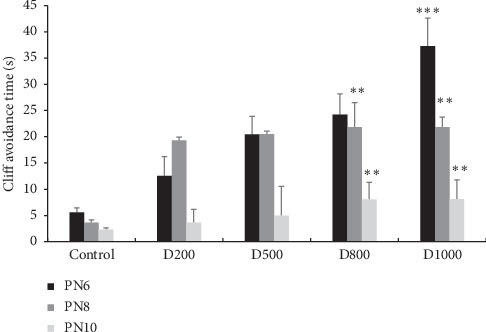
Effects of prenatal treatment with *Trigonella foenum-graecum L.* extract during the entire period of gestation on latencies in cliff avoidance test. Results are presented as mean ± SEM. ^*∗∗∗*^*p* <  0.001; ^*∗∗*^*p* < 0.01 vs. control (one-way ANOVA). PN: postnatal day; D: dose.

The cliff avoidance test shows that all pretreated pups take more time to back away from the cliff. Statistical analyses reveal a significant difference in comparison with control at D800 on the PN8 and PN10 (*p* < 0.01) and at D1000 on the sixth, eighth, and tenth day-old mice (*p* < 0.001 for 6^th^ day-old mice; *p* < 0.01 for 8^th^ and 10^th^ day-old mice) (Figure 4).

#### 3.3.4. Jumping Dnown with Choice Test

**Figure 5:**
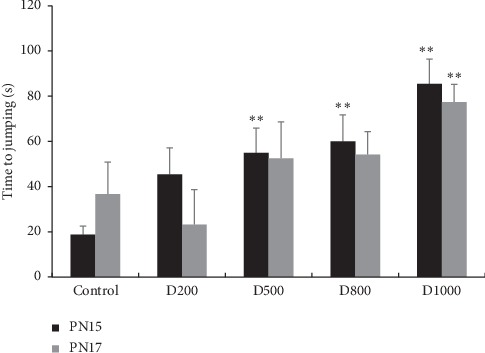
Effects of prenatal treatment with *Trigonella foenum-graecum L.* during the entire period of gestation on latencies in latency to jumping in cage test of 15^th^ and 17^th^ day-old mice. Results are presented as mean ± SEM. ^*∗*^*p* < 0.05; ^*∗∗*^*p* < 0.01 vs. control (one-way ANOVA). PN: postnatal day; D: dose.

Latency to jumping in cage appeared significantly greater in groups receiving treatment at 500, 800, and 1000 mg/kg when compared to the control group, on the 15^th^ postnatal day-old mice (*p* < 0.01). Moreover, for groups treated with 1000 mg/kg/day, this effect is significant for both 15^th^ and 17^th^ postnatal day-old mice (*p* < 0.01) (Figure 5).

In parallel, for the choice between home and empty cage, all treated pups have shown a lower preference for home cage. In fact, more than half of the treated pups have shown a preference for the empty cage on the 15^th^ day-old mice and nearly three quarters for the 17^th^ day-old mice (Table 5).

#### 3.3.5. Rotarod Test

**Figure 6:**
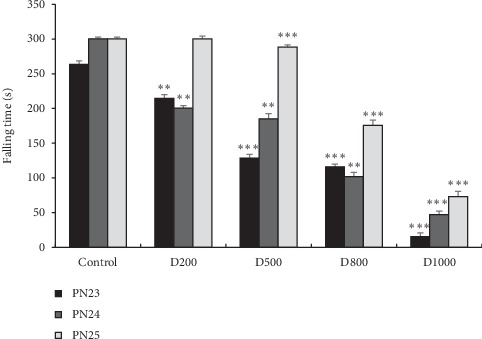
Effects of prenatal treatment with *Trigonella foenum-graecum L.* during the entire gestational period on latencies in falling time in rotarod test of 23, 24, and 25 postnatal days. Results are presented as mean ± SEM. ^*∗∗∗*^*p* <  0.001; ^*∗∗*^*p* < 0.01 vs. control (one-way ANOVA). PN: postnatal day; D: dose.

As it is represented in Figure 6, rotarod test has demonstrated a significant decrease of latencies for falling off the rod in comparison with control, especially at 500, 800, and 1000 mg/kg (*p* < 0.01, at PN24, for 500 and 800 mg/kg; *p* < 0.001, at PN23 and PN25, for 500, 800, and 1000 mg/kg). With the dose of 200 mg/kg, the latency time reduction is significant only at PN23 and PN24 day-old mice (*p* < 0.01) (Figure 6).

### 3.4. Phytochemistry Study

#### 3.4.1. Phytochemical Screening

LeGFS screening indicated the presence of flavonoids, tannins, saponins, anthocyanins, leucoanthocyanins, and the absence of quinones, terpenes, and steroids.

#### 3.4.2. High-Performance Liquid Chromatography Analysis of LeGFS and LeUGFS

**Figure 7:**
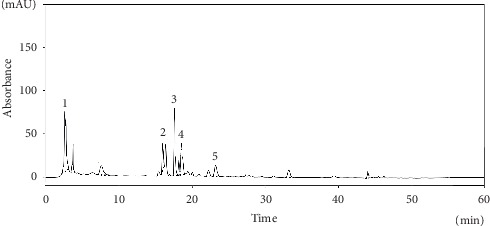
HPLC chromatogram for the main phenolic compounds identified in the aqueous extract of the germinated seeds of *Trigonella foenum-graecum L.*. 1: gallic acid; 2: caffeic acid; 3: syringic acid; 4: tyrosol; 5: rutin.

**Figure 8:**
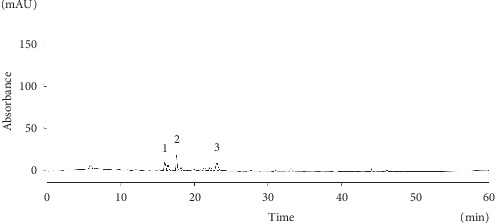
HPLC chromatogram for the main phenolic compounds identified in the aqueous extract of the ungerminated seeds of *Trigonella foenum-graecum L.* (LeUGFS). 1: caffeic acid; 2: syringic acid; 3: rutin.

HPLC analysis of lyophilized aqueous extract of ungerminated (LeUGFS) and germinated fenugreek seeds (LeGFS) (Figures 7 and 8) (Table 6) shows the presence of rutin, caffeic, and syringic acids in both extracts, with a small increase in their concentrations in germinated seeds, noticeably for syringic acid (15.74 mg EGA/100 g DM). Furthermore, sprouted fenugreek seeds show the presence of syringic acid had the highest concentration followed by caffeic acid with a concentration of 13.43 mg EGA/100 g MS, then rutin with a concentration of 13.18 mg EGA/100 g MS, with the presence of gallic acid and tyrosol with concentrations of 12.99 mg EGA/100 g DM and 12.74 mg EGA/100 g DM, respectively. This analysis demonstrates a clear augmentation of total phenolic compounds' concentrations in sprouted seeds in comparison with ungerminated seeds.

## 4. Discussion

The investigation of the side effects of medicinal plants is highly required for safe and appropriate use of herbal drugs. Although fenugreek is one of the oldest and most commonly used medicinal plants and while considering that it has benefited from numerous studies investigating its various pharmacological effects, the teratogenic activity of this herb has not been commonly known until recent years. In fact, some studies revealed a big impact of the seeds' extracts on rodents' reproduction including fertility and teratogenicity [21, 24, 25].

For the fenugreek sprouts, the majority of published studies have focused on its beneficial effects such as antidiabetic activity [29], antiobesity effect [30], and antioxidant action [31]. However, no studies have been carried out about the sprouts' side effects on reproduction even if there are multiple shreds of evidence about the teratogenic and antifertilizing activities of dormant seeds.

Concerning the toxicity of sprouted seeds, Salawu et al. [32] showed that fenugreek powder administered intragastrically to mice and albino rats of both sexes failed to induce any signs of toxicity or mortality up to a maximum practical dose of 2 and 5 g/kg body weight, respectively. In line with previous studies, Omi et al. [29] have shown safe use of sprouted fenugreek aqueous extract administered orally to albino rats. Consequently, in terms of acute toxic effect, the germination of fenugreek seeds does not affect their level of toxicity. These findings are consistent with our results about the maternal toxicity. In fact, neither toxic symptoms nor mortality was shown on treated females during the entire period of pregnancy.

Nevertheless, for the developmental toxicity, the comparison of this side effect between sprouted and dormant seeds has revealed some differences. Germinated seeds provoke abortion in mice at 500 mg/kg and 1000 mg/kg whereas ungerminated seeds failed to induce any abortion in mice at the same doses [24]. This observation let us suppose that germination may induce the synthesis or increase the concentration of one or many ingredients, which are abortive for female pregnant mice. The comparison between HPLC chemical analyses of dormant and sprouted seeds' aqueous extract shows the increase of phenol contents by the process of germination. This finding allows us to suppose that the abortive effect of sprouted seeds could be related to the increase of phenol contents.

Previous research studies as well as our finding about the maternal toxicity of fenugreek show that the plant decreases significantly the female rodent fertility regardless of the fact that it is germinated or not. The study carried out by Kassem et al. [21] shows that feeding rabbits a diet containing 30% of fenugreek seeds provokes a significant reduction in fertility with a decrease of fetal and placental weights at the 20^th^ day of gestation. A similar conclusion was reached by Khalki et al. [24], who demonstrated that aqueous seed extract of fenugreek has a negative impact on the reproductive performance of female mice. Nevertheless, when comparing our results with those of Khalki et al. [24], we see that sprouted seeds have a higher effect than ungerminated seeds at lower doses; for example, the delivery index, which is constant for all doses for seeds, starts to decline at 500 mg/kg for germinated seeds. This observation might be related to the process of sprouting which increases the rate of secondary metabolites as it is shown by the study of Jain [31].

For the pups, the viability and lactation indexes are severely reduced by the prenatal exposition to LeGFS; nonetheless, dormant seeds do not affect the offspring viability [24]. Besides, the pups' growth is significantly reduced at low doses in comparison with high doses. However, when comparing our results to those of Khalki et al. [24], it must be pointed out that prenatal exposition to aqueous extract of nongerminated fenugreek seeds provokes a significant reduction of mice body weight at 500 and 1000 mg/kg until the 28^th^ day-old mice. In the same way, a study conducted by Khan and Khosala [30] has shown a significant decrease in body weight in diabetic patients after being treated with a low dose of 25 mg of sprouted seeds twice on a daily basis. Contrary to this finding, high doses seem to increase the body weight; this may be related to an increase in enzyme concentration, which stimulates the nutrition.

The effects of LeGFS on pups' morphological traits seem to be limited to the following: a delay in the appearance of hair, the opening of eyes, the opening of auditory canals, and the eruption of incisors in mice. For seeds, the extract induces many facial external malformations in mice offspring such as an auricular aplasia, a bump on the head, and a median cleft of the lower lip [24].

The assessment of sensorimotor functions in prenatally exposed mice to LeGFS shows a significant perturbation of vestibular reflex, social behavior with sensorial maturation, and motor coordination as it is shown by the surface righting reflex test, cliff avoidance test, jumping down with choice test, and rotarod test, respectively. Compared to our previous study, it is clear that ungerminated seeds affect also the motor skills in mice; this finding was strongly confirmed by recording the extracellular motor output in the spinal cord isolated from newborn pups. The registered frequency of spontaneous activity and fictive locomotion was significantly reduced by the prenatal exposition to fenugreek seeds' extract [26].

HPLC analysis of aqueous extract has shown the presence of phenolic compounds such as gallic acid, syringic acid, tyrosol, and flavonoids. For the flavonoids, our results tie well with previous studies where the exposition of zebrafish throughout embryologic development to different flavonoid compounds causes mortality and adverse effects on the morphology and behavior. Moreover, the use of estrogenic receptors' antagonist does not inhibit the developmental toxicity of those products, demonstrating that this side effect is not related to the estrogen's classic mode of action, but to the phenol itself [33]. Regarding the abortive effect in mice, this does seem to depend on the placenta formation, considering the fact that Kassem et al. [21] noticed a significant decrease of placenta weight in rabbits prenatally exposed to fenugreek seeds. In fact, the exposition to phenolic compounds may disrupt the formation of placenta, which is a fetal-maternal barrier that provides the fetus with nutrients necessary for its development. The blockage of vessels' growth caused by antiangiogenic activity could be a key to understand this spontaneous pregnancy failure. This assumption is supported by numerous studies demonstrating that rutin and other phenolic compounds are known to be antiangiogenic substances [34–36].

## 5. Conclusion

Our work casts a new light on the abortive, antifertilizing, and teratogenic activities of fenugreek in mice. We found that maternal exposure to germinated seeds induces a spontaneous abortive activity in mice and elicited adverse effects on offspring, such as the perturbation of motor skills and behavior. As we have argued previously the comparison of germinated and nongerminated seeds of fenugreek revealed an increase in phenolic compounds which is the result of seed germination, the presence of these compounds could be the cause of this reproductive toxicity in mice. Further research is needed to confirm this novel finding and investigate the association between the presence of phenolic compounds and developmental toxicity. Nonetheless, on this basis, we can conclude that the consumption of sprouted fenugreek should be avoided during pregnancy.

## Figures and Tables

**Table 1:** Effect of LeGFS on abortion, fertility, and body weight of pregnant mice.

	Control	D200	D500	D800	D1000
Females with positive vaginal plug	8	8	8	8	8
Number of pregnant mice	8	7	7	6	6
Deliverance index (%)	100	100	57.14	33.33	33.33
Number of aborted mice	0	0	3	4	4
Pregnancy index (%)	100	87.50	87.50	75.00	75.00
Abortion index (%)	0	0	42.85	66.66	66.66
Abortion days	—	—	17 ± 0.40^*∗∗∗*^	16 ± 0	15 ± 0.62

Results are presented as mean ± SEM. ^*∗∗∗*^*p* <  0.001 vs. control (ANOVA test). DG: day of gestation; D: dose (mg/kg).

**Table 2:** Effect of LeGFS on reproduction parameters of mice mothers.

	Control	D200	D500	D800	D1000
Number of mice mothers	8	8	8	8	8
Deliverance index (%)	100	100	57.14^*∗∗∗*^	33.33^*∗∗∗*^	33.33^*∗∗∗*^
Viability index (%)	100	89.47	70.37^*∗∗∗*^	82.35^*∗∗∗*^	75.00^*∗∗∗*^
Lactation index (%)	100	89.47	59.25^*∗∗∗*^	70.58^*∗∗∗*^	45.83^*∗∗∗*^

Results are presented as mean ± SEM. ^*∗∗∗*^*p* <  0.001 vs. control (ANOVA test). D: dose (mg/kg).

**Table 3:** Effect of LeGFS on offspring's body weight.

Body weight (g)					
	Control	D200	D500	D800	D1000
PN0	1.60 ± 0.04	1.05 ± 0.03	1.20 ± 0.30	1.43 ± 0.33	1.47 ± 0.05
PN3	2.39 ± 0.05	1.42 ± 0.06^*∗∗∗*^	1.66 ± 0.05^*∗∗∗*^	1.77 ± 0.06^*∗∗∗*^	2.25 ± 0.17
PN6	3.15 ± 0.07	2.01 ± 0.13^*∗∗∗*^	2.4 ± 0.08^*∗∗∗*^	3.10 ± 0.09	3.15 ± 0.22
PN9	3.96 ± 0.09	2.74 ± 0.19^*∗∗∗*^	3.41 ± 0.16	3.54 ± 0.11	4.10 ± 0.29
PN12	4.46 ± 0.16	3.91 ± 0.43	4.01 ± 0.07	4.45 ± 0.10	4.75 ± 0.37
PN15	5.05 ± 0.16	4.16 ± 0.05	5.20 ± 0.14	5.61 ± 0.36	5.76 ± 0.44^*∗*^
PN18	5.52 ± 0.20	4.79 ± 0.16	5.56 ± 0.13	5.68 ± 0.62	5.87 ± 0.51^*∗*^
PN21	6.12 ± 0.13	5.41 ± 0.26	6.69 ± 0.11	6.39 ± 0.72	7.18 ± 0.60^*∗*^

Results are presented as mean ± SEM. ^*∗*^*p* < 0.05, ^*∗∗∗*^*p* <  0.001 vs. control (ANOVA test). PN: postnatal day, D: dose (mg/kg).

**Table 4:** Effect of LeGFS on the body development of offspring.

	Control	D200	D500	D800	D1000
Incisor eruption	PN8 ± 0	PN9 ± 0^*∗∗∗*^	PN9 ± 0.1^*∗∗∗*^	PN9 ± 0.1^*∗∗∗*^	PN9 ± 0^*∗∗∗*^
Appearance of hair	PN6 ± 0	PN7 ± 0.24^*∗∗∗*^	PN7 ± 0.20^*∗∗∗*^	PN8 ± 0.24^*∗∗∗*^	PN8 ± 0.20^*∗∗∗*^
Opening of auditory canal	PN12 ± 0	PN13 ± 0.20^*∗∗*^	PN13 ± 0.20^*∗∗*^	PN13 ± 0.24^*∗∗∗*^	PN13 ± 0.24^*∗∗∗*^
Eye opening	PN14 ± 0	PN14 ± 0.44	PN14 ± 0.54	PN15 ± 0.40^*∗∗∗*^	PN16 ± 0.51^*∗∗∗*^

Results are presented as mean ± SEM. ^*∗∗*^*p* < 0.01, ^*∗∗∗*^*p* <  0.001 vs. control (ANOVA test). PN: postnatal day; D: dose (mg/kg).

**Table 5:** The percentage of mice that choose the home cage.

	Control (%)	D200 (%)	D500 (%)	D800 (%)	D1000 (%)
PN15	100	80	71.42	66.66	41.66
PN17	100	60	57.14	36.36	37.55

PN: postnatal day; D: dose (mg/kg).

**Table 6:** Concentrations of the main phenolic compounds identified in the LeGFS and LeUGFS.

		Gallic acid	Caffeic acid	Syringic acid	Tyrosol	Rutin
Concentrations (mg·EGA/100 g·DM)	LeGSF	12.99	13.43	15.74	12.74	13.18
	LeUGFS	—	12.43	12.87	—	12.79
Retention time (min)	LeGSF and LeUGFS	3.76	15.98	17.55	18.15	23.15

LeGFS: lyophilized extract of germinated fenugreek seeds; LeUGFS: lyophilized extract of ungerminated fenugreek seeds.

## Data Availability

The data used to support the findings of this study are available from the corresponding author upon request.

## Abbreviations

HPLC: High-performance liquid chromatography
LeGFS: Lyophilized extract of germinated fenugreek seeds
LeUGFS: Lyophilized extract of ungerminated fenugreek seeds
PN: Postnatal day
RPM: Rotation per minute
D: Dose
LD50: Lethal dose 50
LDL: Low-density lipoprotein
HDL: High-density lipoprotein.
